# Age- and gender-specific characteristics of the resting-state brain activity: a magnetoencephalography study

**DOI:** 10.18632/aging.103956

**Published:** 2020-11-04

**Authors:** Hideyuki Hoshi, Yoshihito Shigihara

**Affiliations:** 1 Precision Medicine Centre, Hokuto Hospital, Obihiro-shi, Hokkaido, Japan

**Keywords:** gender difference, magnetoencephalography (MEG), normative study, oscillations, resting-state activity

## Abstract

Aging and gender influence regional brain activities. Although these biases should be considered during the clinical examinations using magnetoencephalography, they have yet to be standardized. In the present study, resting-state magnetoencephalography data were recorded from 54 healthy females and 48 males aged 22 to 75 years, who were controlled for cognitive performance. The regional oscillatory power was estimated for each frequency band (delta, theta, alpha, beta, low-gamma, and high-gamma) using the sLORETA-like algorithm and the biases of age and gender were evaluated, respectively. The results showed that faster oscillatory powers increased with age in the rostral regions and decreased in the caudal regions, while few slower oscillatory powers changed with age. Gender differences in oscillatory powers were found in a broad frequency range, mostly in the caudal brain regions. The present study characterized the effects of healthy aging and gender asymmetricity on the regional resting-state brain activity, with the aim to facilitate the accurate and efficient use of magnetoencephalography in clinical practice.

## INTRODUCTION

Recent developments in neuroscience have allowed us to apply neuroimaging techniques in clinical medicine [1, 2]. Clinical neuroimaging techniques fall into two categories: structural and functional neuroimaging. The indications of the former, such as magnetic resonance imaging (MRI), and computer tomography (CT) are already well established and widely used for trauma and organic diseases, such as neurovascular diseases [3], and tumors [4]. In contrast, the clinical indications of functional neuroimaging techniques, such as functional magnetic resonance imaging (fMRI), electroencephalography (EEG), and magnetoencephalography (MEG) have gradually come into use, for disorders such as epilepsy [5], and pre-surgical evaluation of the eloquent cortex [6, 7] of patients undergoing neurosurgical treatments. The measurement methods of functional neuroimaging are subdivided into two categories: event-related (i.e., evoked) and resting-state (i.e., spontaneous) recordings [8]. Although the former is useful and important for pre-surgical mapping of the eloquent cortex [6, 7], it can be used only for patients who are able to understand given tasks. In clinical setting, the latter task-free resting-state measurement is a valuable examination, since patients in any state (e.g., coma, disoriented, and irritated) can be scanned safely and efficiently. As such, it is used for diagnosing various diseases, such as dementia / neurodegenerative diseases [9], brain tumors [10], and other neurological disorders [11–13]. There are two major types of functional neuroimaging methods used to measure the resting-state condition: EEG/MEG, which measures the electrophysiological oscillation of neurons [14], and fMRI, which is a neurovascular coupling-based method [15]. EEG/MEG captures more dynamic neural activities than does fMRI (EEG/MEG has a better temporal resolution and can provide oscillatory power information in wider range with better sensitivity). Furthermore, from a clinical perspective, EEG/MEG has two advantages over fMRI; (1) MRI scans have potential risks. Patients may occasionally bring metallic items into the scanner unintentionally which is highly dangerous in the strong magnetic field produced by the MRI scanners. In case of emergency, it takes up to 10 seconds to abort MRI scan to remove the patient if needed. Moreover, patients often dislike MRI scans as the device generates loud noises [16]. Conversely, EEG/MEG lacks all this potential risks and drawbacks. (2) fMRI is based on blood-oxygen-level-dependent (BOLD) signal changes, which is affected by various factors, such as medications and cardiovascular diseases [17, 18]. Older individuals often suffer from certain conditions where prescribed medications are required (e.g., calcium channel blockers for hypertension) which can affect fMRI data. Although BOLD signal change based on the assumption of neurovascular coupling [19], it remains unclear whether the technique can capture age-related changes [17]. In contrast, EEG/MEG measures neural activity directly, thus it is independent of these assumptions.

EEG and MEG have comparable spatial resolution [20], however, some studies have reported that MEG spatial resolution is superior to that EEG [21, 22]. One major bias towards on-scalp EEG signals is skull’s electrical conductivity [22, 23] which differs between skull layers [24]. Although there are spatial filtering algorithms which take into account the conductivity bias of EEG source modelling [23], MEG is more advantageous as it is free from such biases. In particular, MEG can provide straightforward results when the study involves participants from various backgrounds (e.g., different age and genders), which in turn, the skull conductivities would vary based on the participants. Another advantage of MEG is that it is electrode- and gel-free and has short preparation period. Taken together, for the purpose of clinical assessment / screening, resting-state functional neuroimaging using MEG is both effective and sensitive to detect pathological characteristics of brain activities. Resting-state MEG recording is used during daily examination in our institution; over 700 scans were performed at our two MEG sites in 2018.

Resting-state EEG/MEG represents brain activities in terms of the amplitude or the power of neural oscillations. The power changes with physiological aging, and there is an asymmetricity between females and males. The review [25] summarized the effect of age on EEG signals as (1) an increase in the fast oscillatory powers (beta and gamma) mainly in the rostral region, and (2) a decrease in the slow oscillatory powers (delta, theta, and alpha) mainly in the caudal region (please see [25–27], for reviews). More recently, multiple studies have refined these findings, and it is now generally agreed that physiological aging causes changes to spectral power profiles; a pronounced power decrease of alpha (8–13 Hz), and a global “slowing”, with increases in power in delta (2–4 Hz) and theta (4–8 Hz) ranges and changes of the topographic locations of these frequency bands [28–33]. Compared to the effects of aging, fewer studies have addressed the effects of gender in this topic. To the best of our knowledge, only one study has directly compared the resting-state EEG spectral profiles between genders [34]; it found that females have a significant increase in their parasagittal mean frequency compared to males. Although these studies revealed the effects of age and gender on EEG/MEG 'signals' (i.e., sensor-level data), their effects on regional brain activities (i.e., source-level data), which are important for clinical use, are not well studied.

Changes in the resting-state spectral power profiles reflect two types of changes in the brain: network-based changes, such as thalamo-cortical network, or functional changes of local neural activities. Slower oscillatory powers are primarily produced by the thalamo-cortical network, while faster oscillatory powers depend on local activities [31, 35–41]. This indicates that deviations of resting-state spectral power profiles from controls reflect malfunctions of brain functions, which can be used as EEG/MEG biomarkers for age-associated (and/or gender-related) functional disorders [42]. For example, using resting-state MEG data and their spectral profiles, patients with dementia (e.g., Alzheimer’s disease and vascular dementia) and mild cognitive impairments (MCI) can be screened [9, 42–45]. However, for assessing disorders with such biomarkers, clinical references (i.e., control values) are needed. There are various studies regarding resting-state EEG/MEG data of healthy controls, however, the associated references are fewer and the evidence are weaker than what is observed with other neuroimaging modalities, such as MRI and CT. The central purpose and motivation of the present study is to add references to resting-state MEG control data and empower evidence level of the reference data.

Resting-state brain activities can be measure in two different status; with participants’ eyes closed (EC) or open (EO). Previous studies have used either condition, depending on their recording / experimental environments and study purposes [46]. Some studies have assessed the difference of the spectral profiles between EC and EO conditions directly, using MEG recordings of patients or healthy participants with drug intake [47, 48], and EEG recordings of healthy controls [26, 49–52]. As one review described [46], both conditions have methodological pros and cons. For example, more eye blinks (artefacts) are expected in EO condition, whereas eyes’ rolling (also artefacts) are observed in EC condition. While EC data are stable over sessions, participants can easily drift into drowsiness while keeping their eyes closed. EC and EO conditions generate distinctive neural activities [50]. The control data from one condition is not comparable to that of other condition. For providing control data for both recording conditions, we collected data in both conditions. However, we did not assess the quantitative difference between EC and EO conditions.

The primary objective of the present study is to accumulate references to resting-state MEG control data by providing control information (e.g., influence of healthy aging to MEG data) which can be contrasted into clinical data (e.g., influence of unhealthy aging to MEG data). Previous studies lack source-level reference data. Herein, we provide the source-level reference data by studying the effects of healthy aging and gender on single dataset in both EC and EO conditions, to the best of our knowledge, for the first time. For this purpose, we recorded resting-state brain activity using MEG from 54 females and 48 males ranging from 22 to 75 years old, who were cognitively healthy, and evaluated the effects of age and gender on the regional brain activities. The result of the present study makes the resting-state functional neuroimaging robust and reliable in the clinical setting.

## RESULTS

### MMSE-J

To ensure that participants’ cognitive performance was intact, they completed a Mini-Mental State Examination Japanese version (MMSE-J) [53, 54]. The MMSE-J scores ranged between 26 and 30 (30 is the full score). According to the MMSE-J cut-off criteria (usually 23), none of the participants had neurological disorders or cognitive impairment. The details of the scoring parameters are shown in Table 1. All participants got the full score in ‘Orientation to place’, ‘Language’, ‘Repetition’, ‘Reading’ and ‘Copying’ sections; however, 23 participants answered incorrectly in the ‘Attention and calculation’ section and 22 in the ‘Recall’ section. Twenty participants also answered incorrectly in the ‘Writing’ section, where they wrote a greeting message or a single word despite being instructed to write a sentence. Very few participants lost scores in other sections; four participants lost scores in the ‘Orientation to time’ section, three in the ‘Three-stage command’ section and one in the ‘Registration’ section.

**Table 1:** Descriptive statistics of MMSE-J scores.

**MMSE-J section**	***M***	***SD***	**Max**	**Min**	**#Full (102)**
Orientation to time (5)	4.96	0.20	5	4	98
Orientation to place (5)	5.00	-	5	5	102
Registration (3)	2.99	0.10	3	2	101
Attention and calculation (5)	4.67	0.72	5	2	79
Recall (3)	2.75	0.52	3	0	80
Language (2)	2.00	-	2	2	102
Repetition (1)	1.00	-	1	1	102
Three-stage command (3)	2.97	0.17	3	2	99
Reading (1)	1.00	-	1	1	102
Writing (1)	0.80	0.40	1	0	82
Copying (1)	1.00	-	1	1	102
Total score (30)	29.15	1.04	30	26	49

Numbers in brackets indicate a full score of the section. All participants got the same score (full score) for subsections of “Orientation to place”, “Language”, “Repetition”, “Reading”, and “Copying”; *SD*s are not reported for these subsections. *M*, Mean; *SD*, Standard deviation; Max, Maximum score in the section; Minimum score in the section; #Full, number of participants who got a full score in the section.

### Correlations between age and regional MEG oscillatory power

**Figure 1:**
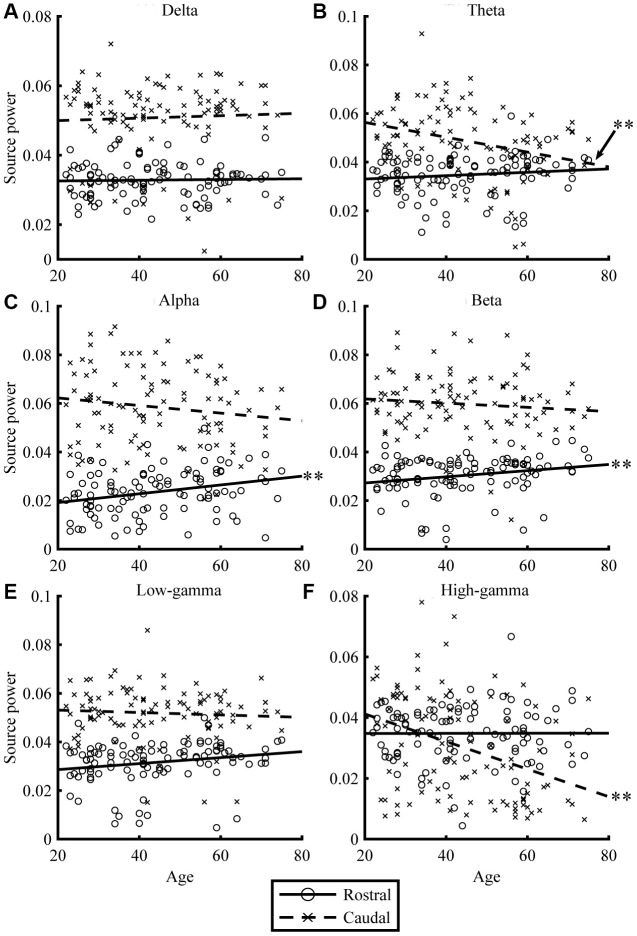
**Relationships between age and regional source power for EC condition.** The scatterplots visualize the relationships between age (x-axis) and regional source power (y-axis) in each frequency band (**A**: Delta, **B**: Theta, **C**: Alpha, **D**: Beta, **E**: Low-gamma, and **F**: High-gamma band) for EC condition. The lines represent linear-fitted (in a least-squares sense) data. Double asterisks (**) indicate significant correlations (please see Table 2 for statistical values).

**Figure 2:**
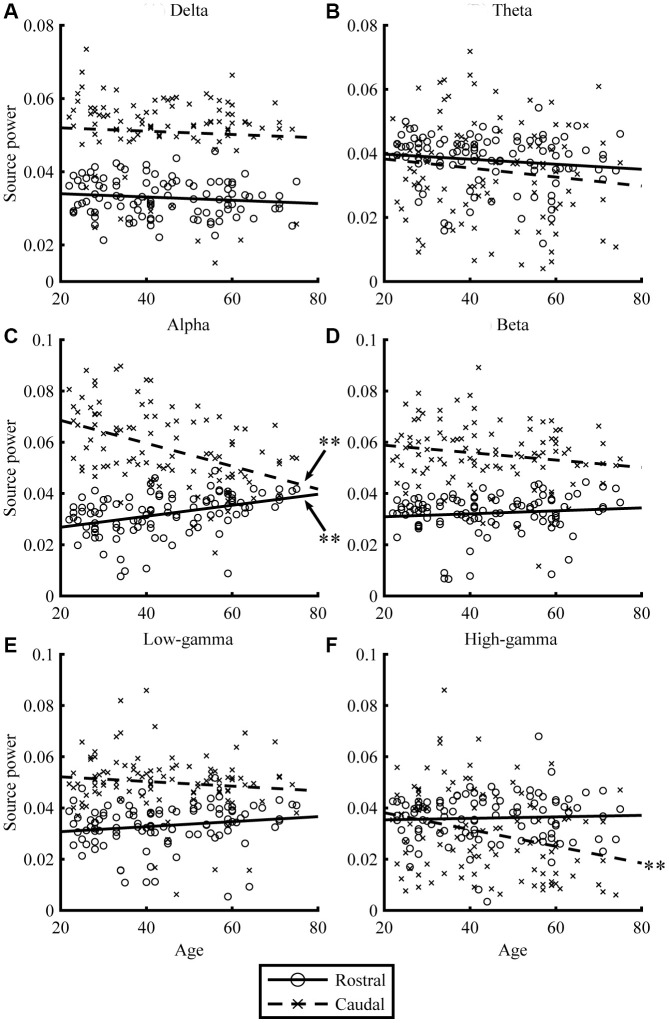
**Relationships between age and regional source power for EO condition.** The scatterplots visualize the relationships between age (x-axis) and regional source power (y-axis) in each frequency band (**A**: Delta, **B**: Theta, **C**: Alpha, **D**: Beta, **E**: Low-gamma, and **F**: High-gamma band) for EO condition. The lines represent linear-fitted (in a least-squares sense) data. Double asterisks (**) indicate significant correlations (please see Table 2 for statistical values).

Resting-state MEG data were acquired for 300 s, for eyes-closed (EC) and eyes-open (EO) conditions. By applying the source-inversion techniques to the MEG data (please see Materials and Methods section for the details), oscillatory powers of the resting-state cortical activities were projected on the source space, for six frequency bands (delta, theta, alpha, beta, low gamma, and high gamma) separately. The correlations between regional oscillatory power and age are shown in Figures 1 and 2, and Table 2. For EC condition, aging significantly reduced caudal theta (*r* = -0.30, *p* = .002) and high-gamma power (*r* = -0.37, *p* < .001), while it significantly increased rostral alpha (*r* = 0.28, *p* = .005) and beta powers (*r* = 0.22, *p* = .025). In EO condition, aging significantly reduced caudal alpha (*r* = -0.44, *p* < .001) and high-gamma powers (*r* = -0.28, *p* = .005), while it significantly increased rostral alpha (*r* = 0.38, *p* < .001) power. Overall, the results for the EC (Figure 1) and EO (Figure 2) conditions were similar. When the correlations were strong, the direction of the relationship was the same, positive or negative, for both conditions (see *r* in Table 2). The relative powers in the rostral region showed positive relationships with age, whereas the caudal regions showed negative relationships. Please note that the significant relationships were present when using nonparametric statistical approaches (please see Supplementary Table 1 in Supplementary Material for details).

**Table 2:** Correlations between age and regional source powers.

		**Delta**		**Theta**		**Alpha**		**Beta**		**Low-gamma**		**High-gamma**	
		***r***	***p***	***r***	***p***	***r***	***p***	***r***	***p***	***r***	***p***	***r***	***p***
(A) EC	Rostral	0.03	0.764	0.12	0.231	0.28*	0.005	0.22*	0.025	0.20	0.045	0.00	0.976
	Caudal	0.05	0.640	-0.30*	0.002	-0.14	0.151	-0.10	0.330	-0.07	0.512	-0.37*	0.000
(B) EO	Rostral	-0.12	0.211	-0.14	0.173	0.38*	< .001	0.11	0.289	0.17	0.097	0.05	0.650
	Caudal	-0.06	0.544	-0.13	0.190	-0.44*	< .001	-0.17	0.090	-0.11	0.261	-0.28*	0.005

Pearson’s correlation coefficients (r) between age and mean source power in each region (Rostral and Caudal), shown for each condition (Eyes-closes and Eyes-open) and frequency band (Delta, Theta, Alpha, Beta, Low-gamma and High-gamma). The *p*-values (*p*) are for testing the hypothesis that there is no significant relationship (null-hypothesis), which were controlled for multiple comparison using FDR method. Asterisks indicate the coefficient is statistically significant after applying FDR correction. *r*, Pearson’s correlation coefficient; *p*, *p*-value; EC, eyes-closed; EO, eyes-open.

### Effect of age on MEG oscillatory power on source space

**Figure 3:**
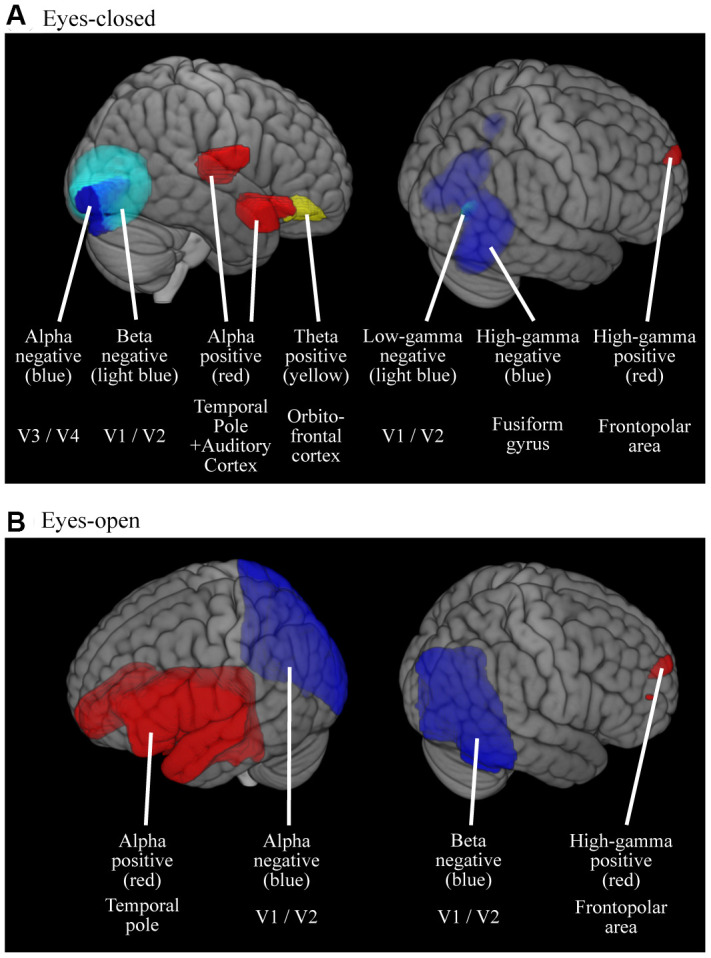
**Cortical regions where the oscillatory powers were significantly predicted by age.** Colored areas represent the clusters where the oscillatory powers were significantly predicted by age, either in positive- or negative-fashion (*p* < 0.05, FWE-corrected). The results were thresholded at alpha level then superimposed on the brains, thus the intensities of color do not mean anything. (**A**) Results for EC condition (**B**) Results for EO condition. M, Males; F, Females.

The effect of age on oscillatory power in source space is visualized in Figure 3 and their peak information is described in Table 3. The results are comparable to those of the correlation analysis. For the EC condition (Figure 3A and Table 3A), theta, alpha, and high-gamma powers were positively related to age in the right fronto-temporal areas (peak locations were in the right orbitofrontal cortex for theta, right temporal pole / auditory cortex for alpha, and right frontopolar area for high-gamma power), whereas alpha, beta, and gamma powers were negatively related to age in the right occipital/posterior temporal areas (peak locations were in right V4 / V3 for alpha, right V1 /V2 for beta, right V1 / V2 for low-gamma, and right fusiform gyrus for high-gamma power). The EO (Figure 3B and Table 3B) condition showed similar trends to those observed in the EC condition, but coarser; alpha and high-gamma powers were positively related to age in a broad area spreading from the frontal to the temporal cortices (peak locations were in left temporal pole for alpha and right frontopolar area for high-gamma power), whereas alpha and beta powers were negatively related to age in a large area covering the occipital cortex (peak locations were in right V1 / V2 for both of alpha and beta powers).

**Table 3:** Summary of the age-related MEG source peak co-ordinates (MNI) and their statitcal significance.

**Condition**	**Age predictor**	**Frequency band**	**LR**	**Cortical area**	**X**	**Y**	**Z**	***t***	***p* (FWE)**
(A) EC	Positive	Theta	R	Fo3 (orbitofrontal cortex)	24	44	-16	3.81	.031
		Alpha	R	Temporal pole	50	18	-22	3.95	.018
			R	Te1.0 (auditory cortex)	50	-14	10	3.93	.018
		High-gamma		Fp1 (frontopolar area)	14	68	22	3.75	.040
	Negative	Alpha	R	V4 / V3	26	-82	-6	4.42	.004
		Beta	R	V1 / V2	4	-68	6	4.57	.002
		Low-gamma		V1 / V2	16	-66	0	3.81	.030
		High-gamma		Fusiform gyrus	32	-56	-4	4.31	.008
(B) EO	Positive	Alpha	L	Temporal pole	-50	10	-14	5.46	< .001
		High-gamma	R	Fp1 (frontopolar area)	12	64	20	3.76	.041
	Negative	Alpha	R	V1 / V2	26	-68	6	5.95	< .001
		Beta	R	V1 / V2	14	-64	2	5.11	< .001

The name of cortical area, MNI coordinates, and relevant statistical values at a peak voxel of SPM (corresponding to Figure 3). (**A**) Results for EC condition (**B**) Results for EO condition. The p-values were corrected for multiple comparisons by the FWE. LR, Left or Right; X, X-coordinate; Y, Y-coordinate; Z, Z-coordinate; *t*, *t*-value; *p*, *p*-value; FWE, Family-wise-error correction; EC, eyes-closed; EO, eyes-open.

### Effect of gender on MEG oscillatory power in source space

**Figure 4:**
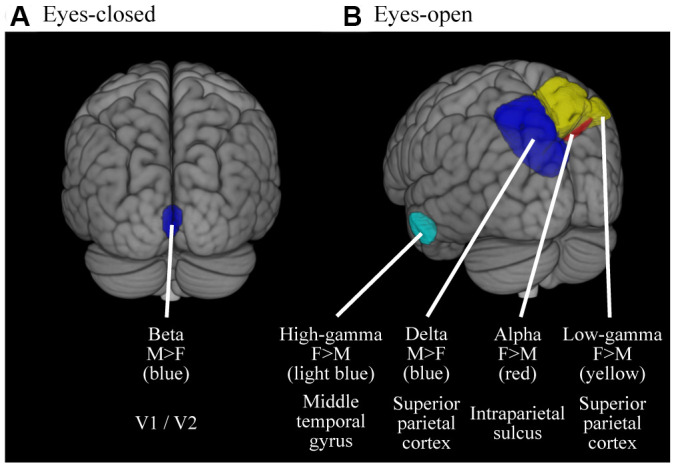
**Cortical regions where the oscillatory powers were significantly different between gender.** Colored areas represent the clusters where the oscillatory powers were significantly different between gender (*p* < 0.05, FWE-corrected). The results were thresholded at alpha level then superimposed on the brains, thus the intensities of color do not mean anything. (**A**) Results for EC condition (**B**) Results for EO condition. M, Males; F, Females.

The gender-specific oscillatory powers are mapped on the source space (Figure 4 and Table 4). Gender differences were found in limited areas. In the EC condition (Figure 4A and Table 4A), the occipital beta power was higher in males than in females (peak location was in bilateral V1 / V2). In the EO condition (Figure 4B and Table 4B), the right parietal alpha (peak location was in right intraparietal sulcus), parietal low-gamma (peak location was in right superior parietal cortex) and left temporal high-gamma powers (peak location was in left middle temporal gyrus) were higher in females than in males; the left parietal delta power (peak location was in left superior parietal cortex) was higher in males than in females.

**Table 4:** Summary of the gender-related MEG source peak co-ordinates (MNI) and their statistical significance.

**Condition**	**Contrast**	**Frequency band**	**LR**	**Cortical area**	**X**	**Y**	**Z**	***t***	***p* (FWE)**
(A) EC	Male > Female	Beta	LR	V1 / V2	0	-88	6	3.71	.032
(B) EO	Female > Male	Alpha	R	Intraparietal sulcus	26	-54	36	3.71	.041
		Low-gamma	R	Area 7A / 7P (Superior parietal cortex)	12	-64	58	4.83	.001
		High-gamma	L	Middle temporal gyrus	-64	-16	-24	3.89	.029
	Male > Female	Delta	L	Area 7A / 7P (Superior parietal cortex)	-14	-64	52	4.35	.009

The name of cortical area, MNI coordinates, and relevant statistical values at a peak voxel of SPM (corresponding to Figure 4). (**A**) Results for EC condition (**B**) Results for EO condition. The p-values were corrected for multiple comparisons by the FWE. LR, Left or Right; X, X-coordinate; Y, Y-coordinate; Z, Z-coordinate; *t*, *t*-value; *p*, *p*-value; FWE, Family-wise-error correction; EC, eyes-closed; EO, eyes-open.

## DISCUSSION

Resting-state functional neuroimaging has gradually come into clinical use. To fully benefit from this technique, it is important to know how healthy aging and gender differences affect the resting-state regional brain activities. To achieve these goals, we collected MEG data from 54 females and 48 males, who were cognitively healthy, and assessed the relationship between regional oscillatory power and age and gender profiles. In the present study, we showed that (1) few slower oscillatory powers changed with age, (2) faster oscillatory powers increased with age in the rostral region of the brain, whereas they decreased in the caudal region, (3) age-dependent oscillatory power changes were more focal in the eye-closed condition than in the eye-open condition, (4) gender differences in oscillatory powers were observed in a broad frequency range (from delta to high gamma), and (5) the gender differences were mostly observed in the caudal brain regions. Males exhibited stronger power in lower frequency in the left hemisphere and females exhibited stronger power in higher frequency in the right hemisphere. These findings corroborated previous results using EEG/MEG and the present study provides additional region-specific information, which is essential for clinical use.

### Age-dependent changes of cortical oscillatory powers

According to previous studies using EEG, there are two major age-dependent changes in neural oscillatory powers, (1) a decrease in slow oscillatory powers (delta, theta, and alpha) mainly in the caudal region (2) an increase in the fast oscillatory powers (beta and gamma) mainly in the rostral region [25–27, 55]. Changes in the resting-state spectral power profiles reflect two types of changes in the brain: network-based changes, or functional changes of local neural activities. Slower oscillatory powers are primarily produced by non-local neural network, such as thalamo-cortical network, while faster oscillatory powers depend on local activities [31, 35–41]. In the following sections, we will discuss these separately.

### Age-dependent changes in low frequencies

Low frequency neural oscillatory powers are primarily produced by non-local neural networks, such as subcortical-cortical and cortico-cortical networks [31, 35–41]. Alpha oscillation is modulated by thalamo–cortical and cortico–cortical interactions, which facilitate/inhibit the transmission of sensorimotor information and the retrieval of semantic information from cortical storage [56–58]. The system is sustained by excitatory activity in the cholinergic brainstem pathway: its function becomes gradually weaker with age, leading to the reduction of alpha power [59, 60]. Theta oscillation is produced by networks between the cortex and subcortical regions such as the hypothalamic and septal regions, and the hippocampus [31, 38].

For slower oscillations, the theta power in the EC condition decreased in the caudal region with age (Figure 1B). The alpha power in the EO condition decreased in the caudal region but increased in the rostral region with age (Figure 2C), which indicates that distinctive systems modulate alpha power in the rostral and caudal regions. Previous studies have consistently found that alpha power is weaker in older individuals [26, 61, 62]. Another study using EEG showed at least two different types of regional alpha oscillatory powers; occipito-parietal and occipito-temporal alphas [63]. This suggests that there are multiple sources of alpha oscillatory powers, which have distinctive age-dependent behaviors [63, 64], which supports our interpretation that age-dependent alpha power changes were distinctive between the rostral and caudal regions. Alpha oscillation plays an important role in processing sensory signals [65], and, since sensory areas are located in the caudal region, decreasing sensory inputs could be one of the reasons why caudal alpha powers were reduced with age. This hypothesis was supported by the fact that age-dependent alpha decrease was significant in the EO condition in which visual system is active and visual input affected brain activities.

Regarding the theta power, it has been demonstrated that older healthy controls (70 ± 7.8 years) exhibit reduced global theta power when compared with younger participants (23 ± 4.8 years) especially in the posterior, midline, and right hemisphere [61]. Another study showed that theta power changed in a quadratic fashion; it decreased marginally between 20 and 40 years of age and then increased slightly after 50 years [66]. A MEG study with 220 participants showed a quadratic change of delta and theta powers in healthy aging participants [67]. Our results were largely consistent with these previous studies. Theta power in the EC condition decreased with age in the caudal region (Figures 1B and 2B), while increased with age in the orbitofrontal cortex (Figure 3A and Table 3). The reduction of hippocampal volume (and / or age-related cognitive impairment) which we have shown in thickness analysis of the MRI data (Supplementary Materials) potentially explains the reduced theta oscillation. However, a previous study has shown that theta power decreased in older healthy controls in comparison with younger participants even with having similar hippocampal volume and cognitive performance [68]. We interpreted that theta power reduction could correspond to the levels of neurogenesis rate. Older adults have fewer new neurons since they recruit new ones with long intervals [69]. The longer interval of neurogenesis in the hippocampus may explain the global theta power reduction. The increased theta oscillatory power in the orbitofrontal cortex among the elderly was consistent with a previous study that showed that a higher theta power at rest in the frontal and parietal regions in healthy older adults is associated with better cognitive function, and is a sign of healthy neurocognitive aging [70]. This would be a good explanation for the results of this study.

Previous studies have found that delta power is weaker among older individuals [55]. A MEG study with 220 participants showed a quadratic change of delta powers in healthy aging participants [67]. The present study did not reveal any age-dependent changes in delta power (Figures 1–3). This could be explained by the fact that delta oscillatory power is mainly related with pathological conditions [71], such as stroke [72, 73] and Alzheimer's disease [74]; therefore, we did not find specific regions which showed age-dependent changes in delta oscillatory power in our healthy volunteers. That enhanced slow oscillatory power could be explained by pathological conditions rather than physiological aging is a useful information for clinical practice.

### Age-dependent changes in high frequencies

Higher frequency oscillations are mainly produced by local neural activities [37, 75–77]. In our study, higher frequency oscillatory powers showed clear age-dependency, which was less evident in lower frequency oscillations (see Figure 1 and 2). Our results showed: (1) age-dependent increases of beta power in the rostral region, (2) age-dependent high-gamma power increases in the orbitofrontal cortex, and (3) age-dependent decreases of all higher oscillatory powers in the caudal region.

Previous studies have shown that beta and gamma fast oscillatory powers increase with age until sixty in cognitively healthy participants [26, 52, 55, 61, 62, 78, 79], which appears to be a favorable sign for preserved intellect among the elderly [25]. In turn, a reduced high frequency power is a not good sign for health; reduced beta power has been shown to be associated with reduced general health among the elderly [25], and a reduction of gamma power has been shown to correspond to a decrease of cortical thickness of the occipital cortex in the elderly [79]. An MEG study with 220 recordings showed a linear increase of high frequency power (mainly beta-1 and beta-2) from childhood until the age of 60 years [67].

In the caudal brain, higher oscillatory powers decreased with age. Gamma oscillatory powers depend on synaptic inhibition mediated by γ-aminobutyric acid (GABA)-containing interneurons [80], and contribute to the visual function [81]. Thus, it is reasonable that fast oscillation and visual function become weaker with healthy aging [82–84]. The GABAergic system plays an important role as an inhibitory control system in the central nervous system. This inhibitory system becomes weaker with age in sensory systems and the hippocampus, while it becomes stronger in prefrontal regions [85–87]. In the rostral brain, higher oscillatory powers increased with age. The increased electrophysiological activities in prefrontal regions is explained by compensation to age-related brain changes [25]. The GABA system is modulated by the dopaminergic (DA) system, which mainly controls the rostral part of the brain [88], and becomes weaker with aging [89–92]. Taken together, it is reasonable that the rostral and caudal brain regions showed different age-dependent gamma frequency profiles. The neurochemical background of beta oscillatory power is controversial, however, it is also related to the DA and GABA systems [93–95], which would account for the observed age-dependent decrease in beta power in parallel with gamma power.

### Gender-specific differences

Both the structure and function of the brain are different between males and females [96, 97]. One prominent structural gender difference is the cortical thickness: globally, females have thicker cortices than males, and females and males have thicker cortices in the left and right hemispheres, respectively [96]. These differences lead to gender differences in neural oscillations. An EEG study showed that females exhibit stronger fast oscillatory powers (i.e., beta 1 and beta 2), while males exhibit stronger slow oscillatory powers (i.e., alpha 2 and theta) [34]. A MEG study has shown that females have a faster alpha frequency, higher beta power, and higher spectral entropy than males [98], and an EEG study has shown that the prefrontal absolute power is higher in females than males [99]. The present study provides more region-specific information about these differences. Most of the differences were observed as laterality in the caudal brain regions. Males showed stronger power in lower frequencies in the left caudal region and females showed stronger power in higher frequencies in the right caudal region. The gender difference in cortical thickness is prominent in the posterior temporal and inferior parietal regions [96]; these regions are functionally connected to occipital areas, where gender differences are reported regarding visual processing [100, 101]. Visual processing and gamma oscillation are linked by the so called ‘visual induced gamma oscillation’ [102], which has been shown to be stronger in females than in males [103]. Cortical thickness is related to gamma power [103] and anatomical differences mainly cause differences in oscillatory power in caudal regions of the brain. Moreover, there are interactions between low (delta, theta, and alpha) and high (beta and gamma) powers [104]. Taken together, this may explain why the oscillatory differences were mainly a laterality in caudal regions of the brain between females and males.

We showed that gender difference was more obvious in EO condition than EC condition (Figure 4). It is known that gender affects information processing through differences in neurotransmitters [105], and that distinctive information processing are working between EC and EO conditions. Information processing in the brain falls into two categories: with and without outside sensory input. Information processing driven by sensory inputs is referred to as 'bottom up' and that without input as 'top-down' processes. Since visual inputs are suppressed during EC condition, the dominance of bottom-up processing is varied between EC and EO conditions. Bottom-up and top-down processing are represented differently in neural oscillatory signals. Bottom-up process is represented by high frequency while the top-down by low frequency, which is dominated by different neurotransmitters: GABAergic neuron serves high (i.e., bottom-up process) and cholinergic neuron serves low frequencies (i.e., top-down process). There are also gender differences in concentrations of GABAergic neurons in some regions, including temporal area [106]. Females show higher frontal cortex cholinergic activity whereas males have higher activity in hippocampus [107]. Taken together, it is possible that EC and EO conditions generated different patterns of gender-specific oscillatory signals, in the caudal part of the brain, which primarily contribute to sensory processing (i.e., bottom-up processing). However, these differences interplay with other factors, such as vigilance, arousal, and attention, whose levels are thought to be different between EC and EO conditions.

### Limitations

The present study provided significant results since it demonstrated the MEG source power at the cortical level with a relatively large population size (102 participants), wide age distribution (age range, 22–75 years) and controlled health status. However, given that the present study aimed to provide a normalized dataset available for clinical research and practice, five limitations can be raised.

First, we recruited the participants from a limited population; our hospital staff members, or their family members, relatives, or friends; thus, some variables, such as economic status and educational levels, will be less varied than the populational level. In this sense, the study was not a true “population study”. However, this limitation brings an advantage to the present study; we could easily confirm that all participants were healthy. This was validated not only in the experimental environment (by using MMSE-J) but also in their life history. As an objective assessment, only MMSE-J was used for controlling participants’ cognitive functions, which is another limitation (e.g., single neuropsychological test cannot ensure that the participants were healthy). Had we recruited participants from the general population and screened them according to the MMSE-J score alone, we would have had faced a risk of recruiting people who were not healthy. We prioritized recruiting 'truly healthy' people to create a reliable reference dataset.

Second, the results should be validated using different source inversion algorithms, such as the minimum norm algorithm (IID in SPM-12; [108]) and the multiple sparse prior algorithm (MSP algorithm in SPM-12; [109]). The goal of the present study is to provide a reference dataset for clinical practice, thus we only used the COH (i.e. sLORETA) algorithm, which is relatively simple, widely recognized, conventional, and non-time-consuming. However, different source inversion algorithms may generate distinctive source patterns [110]. Therefore, the present results should be compared to those of other algorithms for a broader insight regarding the source level effects.

Third, the present study examined the linear effect of age on the source power alone, but its non-linear trends were not considered. Some previous studies have suggested that aging has both linear and non-linear correlations to neuroscientific signals [55, 78]. The non-linear (e.g. quadratic) functions may explain the relationship between age and source power better than the linear function in some conditions.

Fourth, inter-regional relationships, such as connectivity and networks, have not been assessed in this study. This study was motivated by the requirements from clinical examinations: a simpler analysis is favored in daily clinical practice. Thus, we have only studied source-level results of the preprocessed MEG sensor data. Using our method, it takes less than an hour to complete the analysis, and the results are comprehensible for clinicians. However, the cortical network behind the present findings could be useful for future clinical medicine, which should be addressed in the future studies.

Fifth, the quantitative differences between the EC and EO conditions were not examined in the present study. We have reported the results for both conditions, which appear to be distinct. However, it is noteworthy that the displayed differences between the EC and EO conditions (i.e., difference between Figure 1 vs. Figure 2; and Figure 3A vs Figure 3B) are not informative, because the data scales are relative power, and were not statistically compared between EC and EO. Studying the difference between the EC and EO conditions should be assessed in future studies.

## CONCLUSIONS

The present study examined the effects of age and gender on the regional brain activities reflected as the source intensities of resting-state MEG signals. We demonstrated that only some slower oscillatory powers change with age; and that faster oscillatory powers increase in the rostral regions of the brain with age, but decrease in the caudal regions. We found age-dependent oscillatory power changes to be more focal when participants had their eyes open, and that gender differences in oscillatory powers occurred over a broad frequency range. Finally, we observed that gender differences were mostly in the caudal brain region. The scanning and analysis procedures are compatible with a real clinical setting, and the findings helps us to interpret the information of the regional brain activities more effectively and accurately in clinical practice.

## MATERIALS AND METHODS

### Participants

Healthy volunteers were recruited based on the following three criterions. First, all candidates were either staff members of a hospital affiliated with one of the authors, or were their family members, relatives, or friends, who were personally guaranteed by the hospital staff as being healthy and having no disabilities that prevent them from living an ordinary life. Secondly, all candidates were interviewed by a registered clinician to confirm their physical and mental health conditions were within normal range. Third, all participants completed MMSE-J; the cut-off score was defined as >26 (the normal cut-off threshold is 23; [53]). The ‘serial sevens’ version of the MMSE-J was used in the present study. Finally, 102 volunteers (54 females; age range, 22–75 years; mean and SD of age, 44±14.2 years) were enrolled in the study. The breakdowns of participants are described in Table 5. Not all the participants were completely healthy, but were healthy at a passable level; the participants with normal levels of disabilities or diseases, such as mild visual/hearing impairments and hypertension, were included since the present study was aimed at providing a normative dataset that will be used for future clinical examinations. The present study conformed to the ethical principles of the Declaration of Helsinki and was approved by the Ethics Committee of Hokuto Hospital (approval number: 1001). Written informed consent was obtained from each participant during enrolment.

**Table 5:** Distributions of participants.

**Age range**	**Number of Females**	**Number of Males**	**Total**
20-29	12	10	22
30-39	11	9	20
40-49	13	8	21
50-59	10	12	22
60-69	5	6	11
70-79	3	3	6
Total	54	48	102

### Scanning details

Resting-state brain activities were acquired using a 160-channel whole head-type gradiometer (MEG vision PQ1160C, Yokogawa, Kanazawa, Japan) in a magnetically shielded room. The MEG system had a magnetic field resolution of 3 fT/√Hz in the white noise region. The sensing and reference coils in this system are both 15.5 mm in diameter, with a 50 mm baseline and 23 mm of separation between each pair of sensing coils. The recording sampling rate was 1,000 Hz with 200 or 500 Hz low pass filter. Participants were invited to the MEG scan during the daytime (9:00-18:00) which is comparable to the time for normal clinical examination (i.e., hospital hours). They were asked to lie down in supine position with their eyes closed (EC condition; scanning session 1) and eyes open (EO condition; scanning session 2). The data were acquired for 300 s for each condition (i.e., session). They were asked to remain still and stay awake as much as possible during the scans. The participants’ state of vigilance was monitored using a camera in a shielded room, which was double-checked by self-report following the scan (no participants showed / reported obvious changes in their state of vigilance). A screen was placed 30 cm in front of the participants, and a black round fixation point (0.2° diameter of visual angle) was projected from the outside of the magnetically shielded room using a projector (PROPIxx, VPixx Technologies, Saint-Bruno, Canada) through a mirror. Participants watched the fixation point to reduce ocular artefacts during the EO session. Three magnetic marker coils were attached to the skin of the participant’s head (the first two were located at 10 mm in front of the left and right tragus and the third at 35 mm above the nasion) to localize their head positions inside the MEG dewar. Anatomical T1-weighted MRI images were acquired for all participants using a 3.0-T scanner (SIGMA Excite 3.0T, GE Healthcare, Milwaukee, WI) with a standard head coil with three fiducial markers (Medtronic Surgical Navigation Technologies Inc., Broomfield, CO, USA) positioned at the same position of the magnetic marker coils.

### MEG data analysis

To improve the versatility and applicability of the analysis method in the clinical environment, we limited ourselves to try to use as typical and simple analysis methods as we could. Data were analyzed offline using SPM-12 (Wellcome Trust Centre for Neuroimaging, London, UK) and MEAW-system (http://www.hokuto7.or.jp). The data from two scanning sessions (EC and EO) were independently analyzed. For the ease of analysis, continuous MEG signals were divided into 10-s segments. Epochs in which the magnetic signal exceeded 6000 fT were discarded. Data cleaning or artifact corrections (e.g., SSP, PCA, ICA) were not applied to correct for artifacts such as eye-movement, cardiogram, and head motion, since they are not very convenient in the clinical setting; noise reduction procedures usually require longer preparation and data processing times, which increase the physical burden of the patients and the tasks of clinicians, respectively. Additionally, eye-movements were minimized by presenting a fixation point in the EO condition in the present study. A head motion tracking system was unavailable in the MEG system used in the present study. Therefore, we decided not to apply any noise reduction (potential artefacts’ influences on the present results are examined in the Supplementary Materials). Since the experimental environment generated (1) projector artefacts at 120 Hz (due to the minor voltage fluctuations in the projector system) and (2) utility frequency at 50 Hz, a 115-Hz low-pass filter and a 50-Hz band-stop filter (5^th^ order, Butterworth) were applied to the epoched data.

The filtered data were directly used for source-level analyses. To identify the locations of the brain producing the resting-state-induced component, the data was inverted for delta (0–3 Hz), theta (4–7 Hz), alpha (8–12 Hz), beta (13–25 Hz), and gamma (low gamma: 26–40 Hz and high gamma: 41–80 Hz) components. Individual anatomical MRI images were segmented using the unified segmentation algorithm described in [111], which is the default method available in SPM-12. A cortical mesh with 8196 vertices was created using the ‘normal’ mode of the mesh generation function in SPM-12. The coregistration of normalized MRI images and MEG sensor locations was performed using an iterative closest point algorithm [112]. Forward modelling was performed for the whole brain using a single shell model with normalized individual anatomical MRI images. The source inversion was performed using a maximal smoothness algorithm with a spatially coherent sources model (i.e. COH algorithms implemented in SPM-12; [109]), which is similar to sLORETA [113]. The COH algorithm is a commonly used source inversion algorithm and is often used in the clinical environment [114, 115]. Inversion was performed for the bandpass-filtered signal for each frequency band (from delta to high-gamma) without any source priors. A series of Morlet wavelet projectors (i.e., oscillatory powers) were generated summarizing the inverted intensity (i.e., energy) in each trial and each band of interest (from delta to high-gamma). The results were then averaged over trials, which enabled the localization of induced activity that has no phase-locking to the stimulus. The averaged power was projected on the source space then normalized into MNI space, which generated the resulting source images. The source images were smoothed (20 × 20 × 20 mm) and taken to the two types of group-level analysis.

1. To examine the relationships between cortical oscillatory power and age, mean regional powers were calculated for each frequency band. As previous studies have reported that rostral and caudal regions exhibit distinctive power spectral profiles [25–27] and have different neurophysiological bases [88], the relationships were assessed for the rostral and caudal regions separately. We defined the rostral region as a cortical area covering the frontal and temporal cortex, and the caudal region as a cortical area covering the parietal and occipital regions. The extents of the cortices were determined using the WFU PickAtlas (http://fmri.wfubmc.edu/software/pickatlas). The mean power was plotted against the participants’ age for visual inspection, subsequently, Pearson’s correlation coefficient was calculated for each frequency band and each region. The coefficients were tested against the null-hypothesis that there is no significant relationship. Since the plots implied that calculating Pearson’s correlations and following parametric tests may not be the best statistical approach for assessing the data, we have validated the relationships by using non-parametric Spearman's rank correlation coefficient and bootstrap statistics (please see Supplementary Material for the details). The false detection rate (FDR) was controlled using Benjamini and Hochberg method [116].
2. To study the effects of age and gender on the oscillatory power, the source images were compared between genders using a two-sample *t*-test for each frequency band, with the null-hypothesis that there were no-gender differences. The participants’ age was used as a covariate, and its interaction with the gender factor was also included in the model. Both positive and negative effects of age on the source powers were evaluated by building *t*-contrasts with +1 and −1 to the age factor. We did not set any region of interest (ROI) in this analysis; the statistical tests were performed using the source images containing whole-brain data. For each statistical test, the *p*-values were corrected using voxel-wise FWE correction, then the cluster was identified at the extent threshold of *k* > 10 (i.e., a cluster was defined as an area containing more than 10 voxels; Lieberman and Cunningham, 2009). Cortical areas at which the peaks of the *t*-values are located were identified using the SPM Anatomy toolbox [117] (http://www.fz-juelich.de/inm/inm-1/DE/Forschung/_docs/SPMAnatomyToolbox/SPMAnatomyToolbox_node.html). The result was not reported regarding the interaction (age × gender) since no significant clusters were found for the interaction for all frequency bands and conditions (EC and EO).

## Supplementary Material

Supplementary Materials

Supplementary Figures

Supplementary Table 1
