# Supplementary material for: Age- and gender-specific characteristics of the resting-state brain activity: a magnetoencephalography study
Source: Aging (Albany NY). 2020 Nov 4;12(21):21613–37. doi: 10.18632/aging.103956 (PMC7695396; doi:10.18632/aging.103956)
Supplement: Supplementary Materials [file aging-12-103956-s001..pdf]

SUPPLEMENTARY MATERIALS

Supplementary Methods

Effect of noise rejection for sample data

In the present study, MEG data were not cleaned using conventional artefact correction procedures. Here, we examined the potential bias of the artefacts on the results of source inversion by comparing the original (used in the main manuscript) and artefact-cleaned data of a few example participants. We have applied the automated artefact rejection using independent component analysis (ICA) to the MEG data from 10 participants (out of the 102 participants used in the main analysis). The epoched MEG data were decomposed by ICA using FastICA on Fieldtrip [1, 2]. Components correlated with EOG and ECG channels with more than 0.2 in linear correlation coefficient were rejected. The cleaned data were processed with identical procedure described in the main manuscript (see Materials and Methods section). The source images (output from original and artefact-cleaned procedures) were rendered on the template anatomical brain using MRIcroGL software (https://www.mccauslandcenter.sc.edu/mricrogl/) and differences were visually inspected.

ICA results showed that the number of rejected components differed between datafiles (i.e., participants). Out of 10 datafiles, 2 components were rejected in 4 datafiles, while 1 was rejected in other 4 datafiles and there were no rejected in the remaining 2 datafiles. Since rejection of more components bring larger changes on the MEG data, 2 samples (dataset #0005 and #0007) were selected from 4 datasets in which 2 components were rejected. The results were visually inspected for the selected two datasets.

For datafile #0005 (Supplementary Figure 1), the source signals for original and artefact-cleaned source images were mostly overlapped (most regions were colored in yellow). However, artefact-free data estimated source signals in orbitofrontal regions in high-gamma band (green-colored regions in Supplementary Figure 1F), which were not obvious in the original dataset. The results of datafile #0007 showed similar trends (Supplementary Figure 2); the source signals were overlapped mostly between original and cleaned data, except for the high-gamma signals in the orbitofrontal regions (green-colored regions in Supplementary Figure 2F). The results indicated that automated removal of EOG and ECG artefacts had minor influence on the source signals.

Thickness analysis of MRI data

The individual T1-weighted MRI images obtained by 3.0-T scanner (please see Materials and Method section, for details) were used for cortical thickness analysis. ROI-based cortical thicknesses were estimated using Computational Anatomy Toolbox (CAT; http://www.neuro.uni-jena.de/cat/). The mean cortical thicknesses were estimated for caudal and rostral regions (defined by Desikan-Killiany Atlas; [3]). The mean cortical thickness was plotted against the participants' age for visual inspection, subsequently, Pearson's correlation coefficient was calculated between age and each regional cortical thickness. The thickness data were also compared between gender using two-sample *t*-test. Results showed that the ageing reduced cortical thickness in both rostral (r = -0.43, p < .001) and caudal regions (r = -0.62, p < .001). See Supplementary Figure 3 for visualization. There was gender difference in rostral cortical thickness (mean thicknesses were 2.73 mm for females and 2.78 mm for males, r = -2.18, p = .031), but not in caudal thickness (mean thicknesses were 2.27 mm for females and 2.27 mm for males, r = 0.21, p = .834).

Supplementary References

1. Makeig S, Jung T-P, Bell AJ, Sejnowski TJ. Independent Component Analysis of Electroencephalographic Data. Advances in neural information processing systems. 1996; pp. 145–151.

2. Oostenveld R, Fries P, Maris E, Schoffelen JM. FieldTrip: Open source software for advanced analysis of MEG, EEG, and invasive electrophysiological data. Comput Intell Neurosci. 2011;2011:156869. https://doi.org/10.1155/2011/156869 PMID:21253357

3. Desikan RS, Ségonne F, Fischl B, Quinn BT, Dickerson BC, Blacker D, Buckner RL, Dale AM, Maguire RP, Hyman BT, Albert MS, Killiany RJ. An automated labeling system for subdividing the human cerebral cortex on MRI scans into gyral based regions of interest. Neuroimage. 2006; 31:968–80. https://doi.org/10.1016/j.neuroimage.2006.01.021 PMID:16530430
