# Supplementary material for: Age- and gender-specific characteristics of the resting-state brain activity: a magnetoencephalography study
Source: Aging (Albany NY). 2020 Nov 4;12(21):21613–37. doi: 10.18632/aging.103956 (PMC7695396; doi:10.18632/aging.103956)
Supplement: Supplementary Figures [file aging-12-103956-s002..pdf]

SUPPLEMENTARY FIGURES

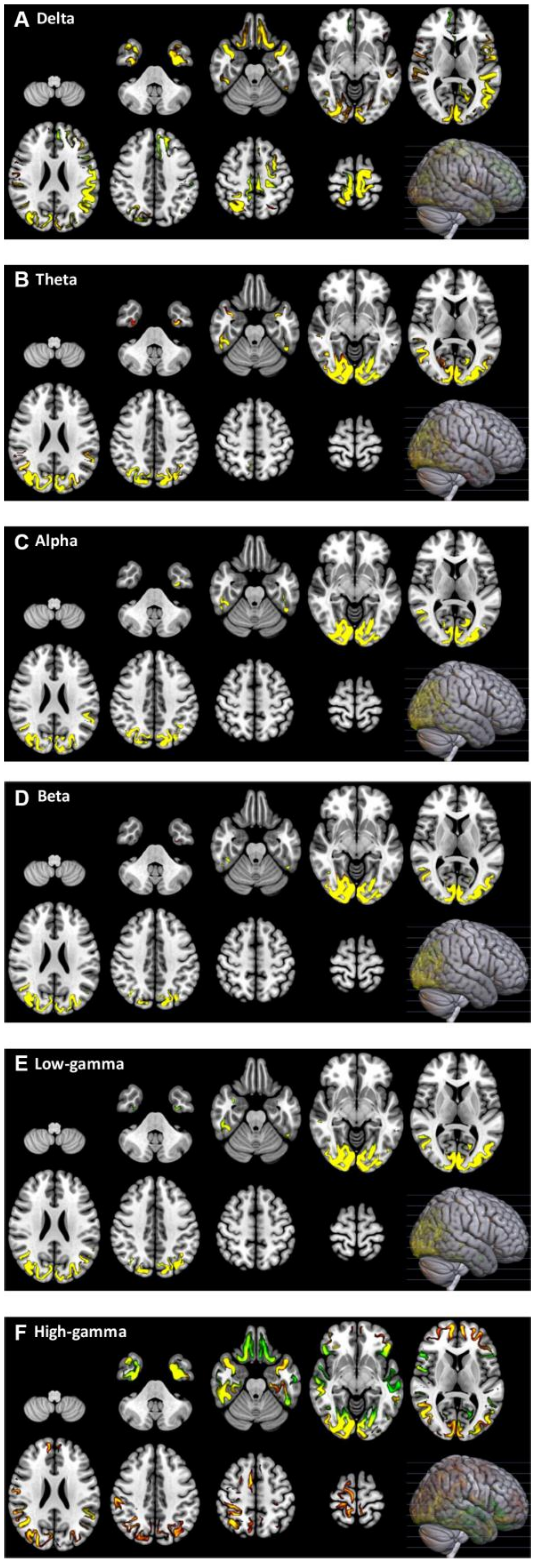

Supplementary Figure 1. Before and after artefact cleaning for data #0005. For each frequency band, the source images generated from original (red) and artefact-cleaned (green) procedures are rendered on the template anatomical brain. In the yellow-colored regions, the source signals of the original and artefact-cleaned results are overlapped. The locations of axial slices are indicated by horizontal lines of 3D brain in the right lower position of each panel.

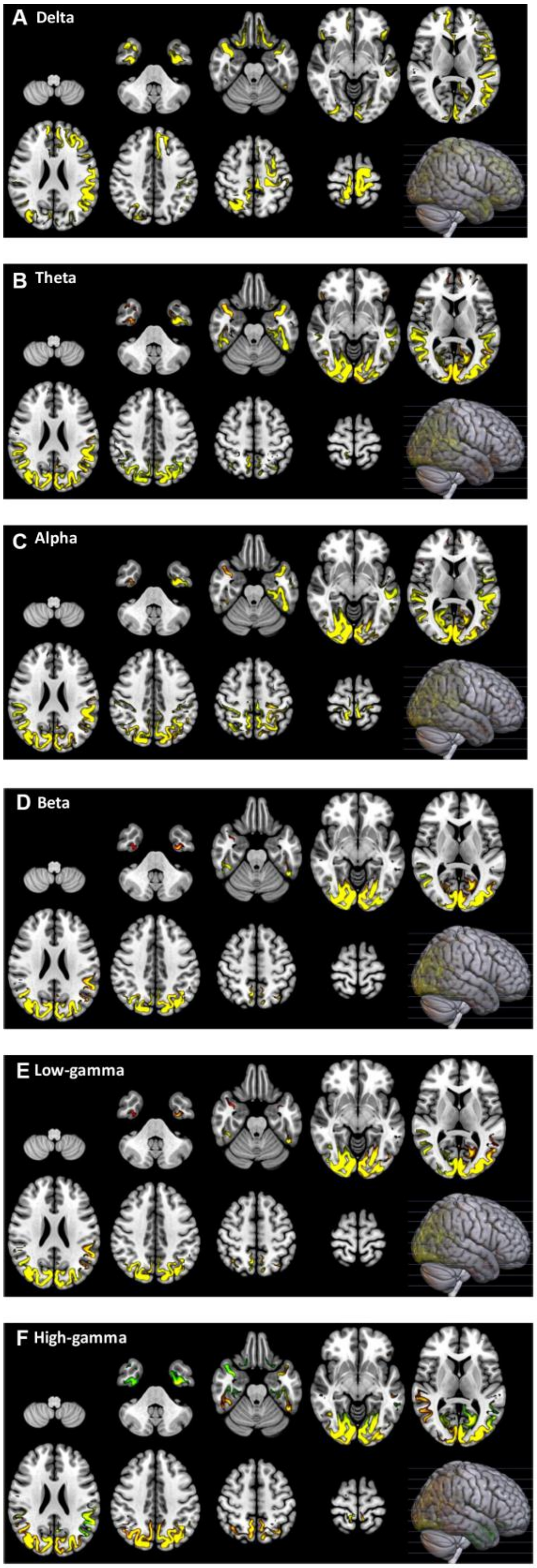

Supplementary Figure 2. Before and after artefact cleaning for data #0007. For each frequency band, the source images generated from original (red) and artefact-cleaned (green) procedures are rendered on the template anatomical brain. In the yellow-colored regions, the source signals of the original and artefact-cleaned results are overlapped. The locations of axial slices are indicated by horizontal lines of 3D brain in the right lower position of each panel.

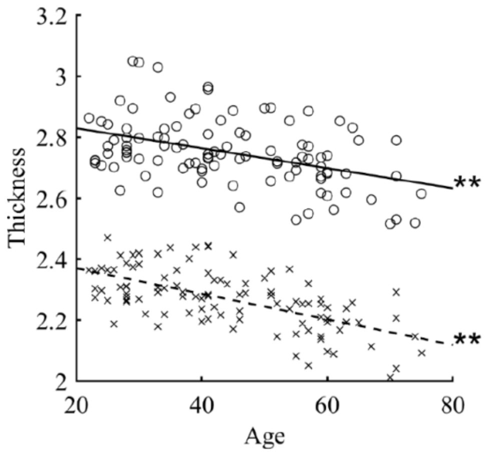

Supplementary Figure 3. Relationships between age and cortical thickness. Circle markers and solid line represent the data for the rostral region, and cross markers and dotted line represent the data for the caudal region.
