# Supplementary material for: Age- and gender-specific characteristics of the resting-state brain activity: a magnetoencephalography study
Source: Aging (Albany NY). 2020 Nov 4;12(21):21613–37. doi: 10.18632/aging.103956 (PMC7695396; doi:10.18632/aging.103956)
Supplement: Supplementary Table 1 [file aging-12-103956-s003..pdf]

SUPPLEMENTARY TABLE

Supplementary Table 1. Nonparametric correlations between age and regional source powers.

		Delta			Theta			Alpha		
		r	***p***	***CI***	***r***	***p***	***CI***	***r***	***p***	***CI***
(A) EC	Rostral	0.05	0.603	-0.14<br>0.24	0.15	0.131	-0.04<br>0.33	0.30*	0.002	0.11<br>0.49
	Caudal	0.03	0.742	-0.16<br>0.24	-0.30*	0.002	-0.44<br>-0.14	-0.11	0.269	-0.30<br>0.09
(B) EO	Rostral	-0.13	0.188	-0.31<br>0.06	-0.18	0.063	-0.36<br>0.01	0.43*	< .001	0.27<br>0.57
	Caudal	-0.10	0.328	-0.29<br>0.11	-0.12	0.219	-0.30<br>0.06	-0.44*	< .001	-0.58<br>-0.29

		Beta			Low gamma			High gamma		
		r	***p***	***CI***	***r***	***p***	***CI***	***r***	***p***	***CI***
(A) EC	Rostral	0.25*	0.013	0.05<br>0.42	0.25*	0.010	0.06<br>0.43	-0.02	0.819	-0.22<br>0.17
	Caudal	-0.07	0.478	-0.25<br>0.11	-0.03	0.796	-0.21<br>0.16	-0.39*	< .001	-0.55<br>-0.20
(B) EO	Rostral	0.10	0.306	-0.10<br>0.29	0.23*	0.019	0.04<br>0.41	0.03	0.777	-0.16<br>0.22
	Caudal	-0.15	0.142	-0.32<br>0.04	-0.06	0.533	-0.26<br>0.13	-0.28*	0.005	-0.45<br>-0.09

Spearman's rank correlation coefficient (*r*) between age and mean source power, results (*p*-value) of the test for no-correlation, and bootstrap confidence interval (*CI*, upper and lower limits are shown for top and bottom row of each cell), shown for each region (rostral and caudal), each condition (Eyes-closes and Eyes-open) and frequency band (Delta, Theta, Alpha, Beta, Low-gamma and High-gamma). In the bootstrap procedure, all participants were resampled with replacement 10,000 times and Spearman's rank correlation coefficient was calculated for each iteration. Confidence interval was calculated using basic percentile method. Asterisks indicate the coefficient is statistically significant after applying FDR correction. *r*, Spearman's rank correlation coefficient; *p*, *p*-value; *CI*, bootstrap confidence interval; EC, eyes-closed; EO, eyes-open.
